# Supplementary material for: Machine Learning for Risk Prediction of Oesophago-Gastric Cancer in Primary Care: Comparison with Existing Risk-Assessment Tools
Source: Cancers (Basel). 2022 Oct 14;14(20):5023. doi: 10.3390/cancers14205023 (PMC9600097; doi:10.3390/cancers14205023)
Supplement: Supplementary file 1 [file cancers-14-05023-s001.zip › cancers-1941770-supplementary.pdf]

cancers MDPI

Supplementary Materials

Machine Learning for Risk Prediction of Oesophago-Gastric Cancer in Primary Care: Comparison with Existing Risk-Assessment Tools

Emma Briggs, Marc de Kamps, Willie Hamilton, Owen Johnson, Ciarán D. McInerney, Richard D. Neal

Table S1. Feature variables. N.B.: Where a binary value has been used for symptoms and lab test results, 0 indicates normal and 1 indicates abnormal.

Category	Variable Name	Definition	Variable Type	ICPC code	Range
**Demographics**	'age_group'	Age group	Binary (0: under 55, 1: 55 and over)	none	{0,1}
**Lab test results**	'chol_sq'	Serum cholesterol (given as squared value)	Float	T34006	[23, 182]
	'abn_low_MCV'	Abnormally low MCV	Binary	A34011 (Full blood count)	{0, 1}
	'abn_low_haem'	Abnormally low haemoglobin	Binary	B34018	{0, 1}
	'abn_hi_plat'	Abnormally high platelet count	Binary	B34005	{0, 1}
	'abn_hi_LFT'	Abnormally high liver function test	Binary	D34008	{0, 1}
	'abn_hi_IM'	Abnormally high inflammatory markers	Binary	B33007	{0, 1}
	'abn_hi_wcc'	Abnormally high white cell count	Binary	A34011 (Full blood count)	{0, 1}
**Symptoms**	'sym_d12_constipation1'	Constipation, first presentation	Binary	D12	{0, 1}
	'sym_a11_chest_pain1'	Chest pain, first presentation	Binary	A11	{0, 1}
	'sym_d01_abdo_pain1'	Abdominal pain, first presentation	Binary	D01	{0, 1}
	'sym_t08_weightloss1'	Weight loss, first presentation	Binary	T08	{0, 1}
	'sym_d21_dysphagia1'	Dysphagia, first presentation	Binary	D21	{0, 1}
	'sym_d21_dysphagia2'	Dysphagia, second presentation	Binary	D21	{0, 1}
	'sym_d84_reflux1'	Reflux, first presentation	Binary	D84	{0, 1}
	'sym_d02_epigastric_pain1'	Epigastric pain, first presentation	Binary	D02	{0, 1}
	'sym_d07_dyspepsia1'	Dyspepsia, first presentation	Binary	D07	{0, 1}

'sym_d07_dyspepsia2'	Dyspepsia, second presentation	Binary	D07	{0, 1}
'sym_d10_nausea_vomiting1'	Nausea/Vomiting, first presentation	Binary	D10	{0, 1}
'sym_d10_nausea_vomiting2'	Nausea/Vomiting, second presentation	Binary	D10	{0, 1}

Table S2. Hyperparameter tuning strategy. Includes list of hyperparameters searched during grid-search cross-validation for fine-tuning models and the corresponding results of each search. 5-fold cross-validation was used for all models. The scoring metric used to select optimal values was mean accuracy.

Model	Hyperparameter	Values searched	Outcome
**Random Forest**	random_grid	{True}	True
	max_depth	{25, 50, 75, None}	75
	max_features	{'auto', 'sqrt'}	'auto'
	min_samples_leaf	{2, 4}	4
	min_samples_split	{2, 5, 10}	5
	criterion	{'entropy'}	'entropy'
	n_estimators	{100, 150, 200, 250, 500}	250
**Support Vector Machine**	C	{0.1, 1, 10, 100}	0.1
	gamma	{0.001, 0.01, 0.1, 1}	1
	kernel	{'rbf', 'sigmoid', 'poly', 'linear'}	'rbf'
**Extreme Gradient Boosted Decision Trees**	min_child_weight	{1, 5, 10}	10
	gamma	{0.5, 1, 2, 5}	5
	subsample	{0.5, 0.8, 1.0}	0.8
	colsample_bytree	{0.5, 0.8, 1.0}	0.5
	max_depth	{3, 4, 5}	4
**Logistic Regression**	solver	{'lbfgs', 'liblinear', 'sag'}	'sag'
	C	{0.01, 0.1, 1, 10, 100, 1000}	0.1

Table S3. Performance for all machine learning based probabilistic classifiers, across a range of thresholds, in comparison with oesophago-gastric cancer risk assessment tool (ogRAT) for prediction of oesophago-gastric cancer incidence, on test dataset. For machine learning models, the classification threshold range is given between 0.3 and 0.8 (in increments of 0.1) to represent best performance and a trade-off between precision and recall which is comparable to that of the ogRAT. ogRAT performance is displayed at the 0.01, 0.02, and 0.03 risk thresholds which are the risk thresholds realistically considered in practice when using the ogRAT.

Classifier	AUROC	Classification threshold	Accuracy	Precision	Recall	F1
**Support Vector Machine (Linear kernel)**	0.869	0.3	0.892	0.760	0.631	0.690
		0.4	0.894	0.827	0.560	0.668
		0.5	0.892	0.850	0.525	0.649
		0.6	0.887	0.878	0.475	0.616
		0.7	0.880	0.887	0.426	0.576

		0.8	0.880	0.903	0.413	0.567
**Support Vector Machine (Radial Basis Function kernel)**	0.800	0.3	0.890	0.753	0.625	0.683
		0.4	0.893	0.789	0.599	0.681
		0.5	0.894	0.814	0.579	0.676
		0.6	0.894	0.836	0.551	0.664
		0.7	0.890	0.850	0.513	0.640
		0.8	0.886	0.870	0.457	0.599
**Logistic Regression**	0.869	0.3	0.889	0.741	0.641	0.688
		0.4	0.894	0.810	0.579	0.675
		0.5	0.892	0.845	0.533	0.654
		0.6	0.887	0.870	0.478	0.617
		0.7	0.881	0.885	0.435	0.584
		0.8	0.880	0.896	0.418	0.570
**Random Forest**	0.861	0.3	0.880	0.681	0.690	0.684
		0.4	0.891	0.757	0.634	0.690
		0.5	0.893	0.819	0.563	0.667
		0.6	0.886	0.859	0.482	0.618
		0.7	0.876	0.915	0.389	0.546
		0.8	0.874	0.917	0.360	0.516
**Bernoulli Naïve Bayes**	0.861	0.3	0.877	0.672	0.658	0.665
		0.4	0.884	0.705	0.640	0.671
		0.5	0.889	0.747	0.610	0.671
		0.6	0.892	0.775	0.587	0.668
		0.7	0.891	0.796	0.550	0.650
		0.8	0.874	0.811	0.427	0.559
**eXtreme Gradient Boosted Decision Trees (‘XGBoost’)**	0.866	0.3	0.886	0.718	0.664	0.690
		0.4	0.892	0.795	0.585	0.674
		0.5	0.893	0.846	0.538	0.657
		0.6	0.886	0.875	0.473	0.614
		0.7	0.880	0.899	0.420	0.572
		0.8	0.879	0.911	0.392	0.548
**Oesophago-gastric Risk-Assessment Tool (ogRAT)**	0.813	0.010	0.873	0.861	0.414	0.559
		0.020	0.869	0.909	0.334	0.489
		0.030	0.866	0.911	0.313	0.466

Feature Contribution Graphs

Below are a series of graphs representing a rough estimation of the 'feature importance' for each model, i.e., an estimation of the relative contribution of each feature to the model risk score.

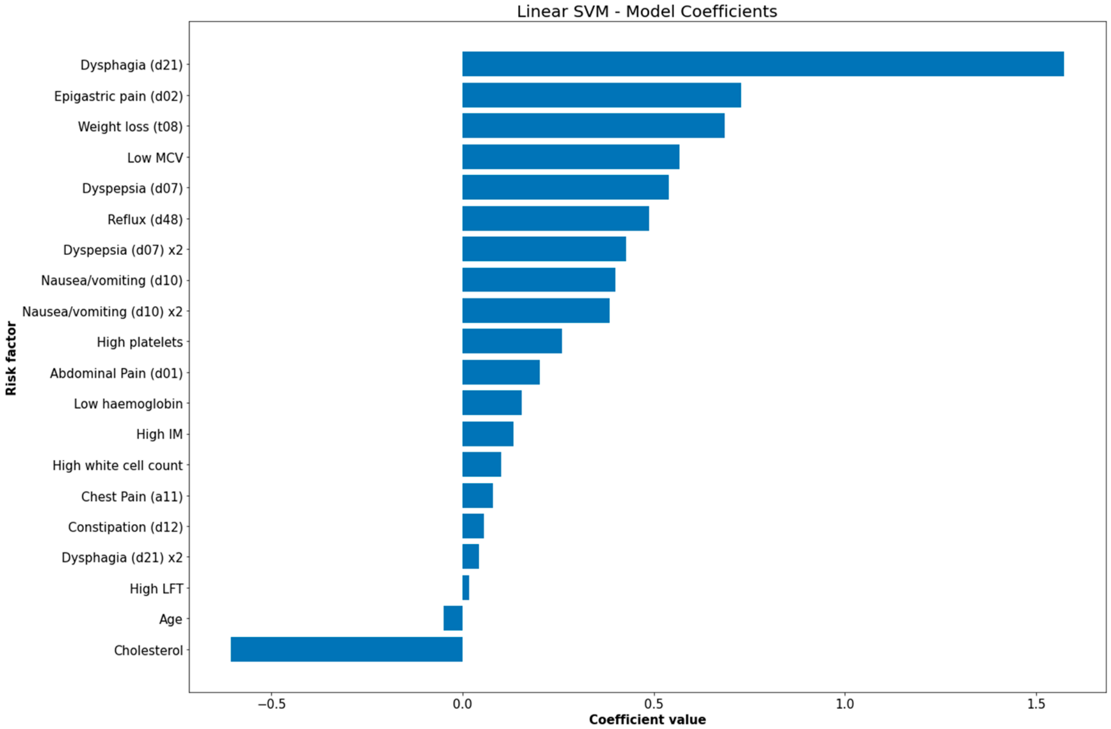

Figure S1. Feature contribution estimation for Support Vector Machine (Linear kernel). Feature contribution values approximated using model coefficients.

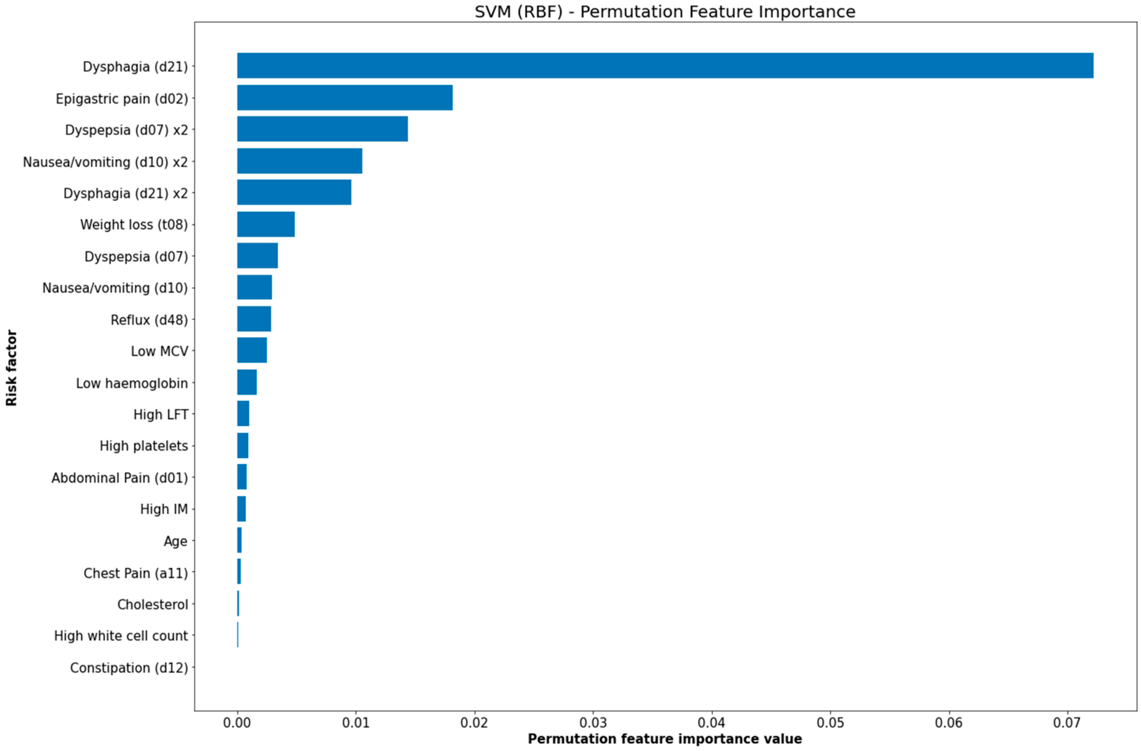

Figure S2. Feature contribution estimation for Support Vector Machine (Radial Basis Function kernel). Feature contribution values approximated using permutation feature importance (i.e., the

mean relative decrease in the model accuracy score when a single feature value is randomly shuffled).

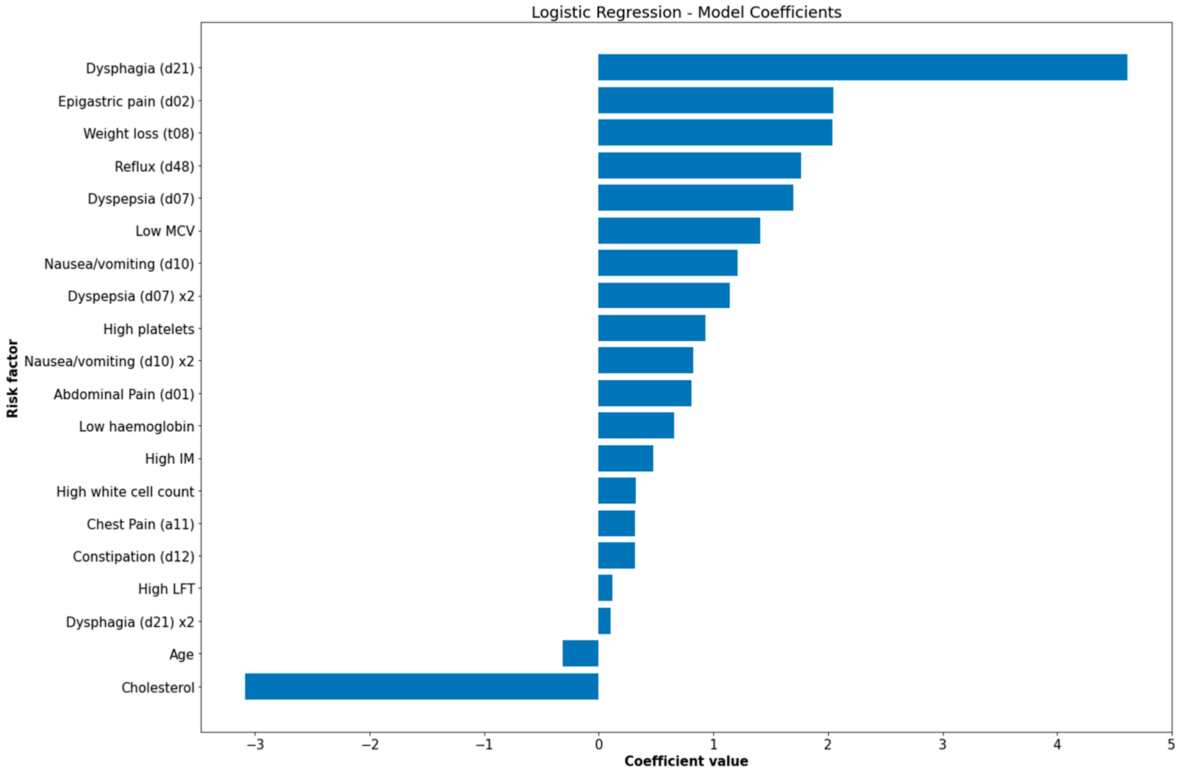

Figure S3. Feature contribution estimation for Logistic Regression. Feature contribution values approximated using model coefficients.

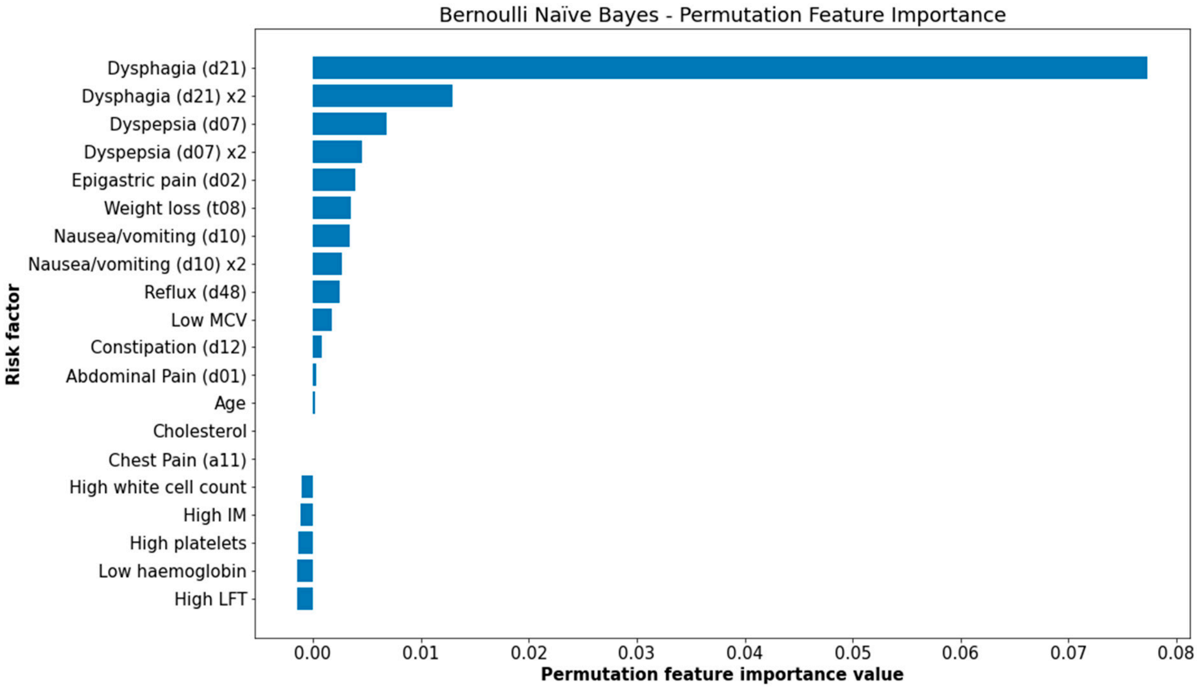

Figure S4. Feature contribution estimation for Naïve Bayes (Bernoulli). Feature contribution values determined using permutation feature importance (i.e., the mean relative decrease in the model accuracy score when a single feature value is randomly shuffled).

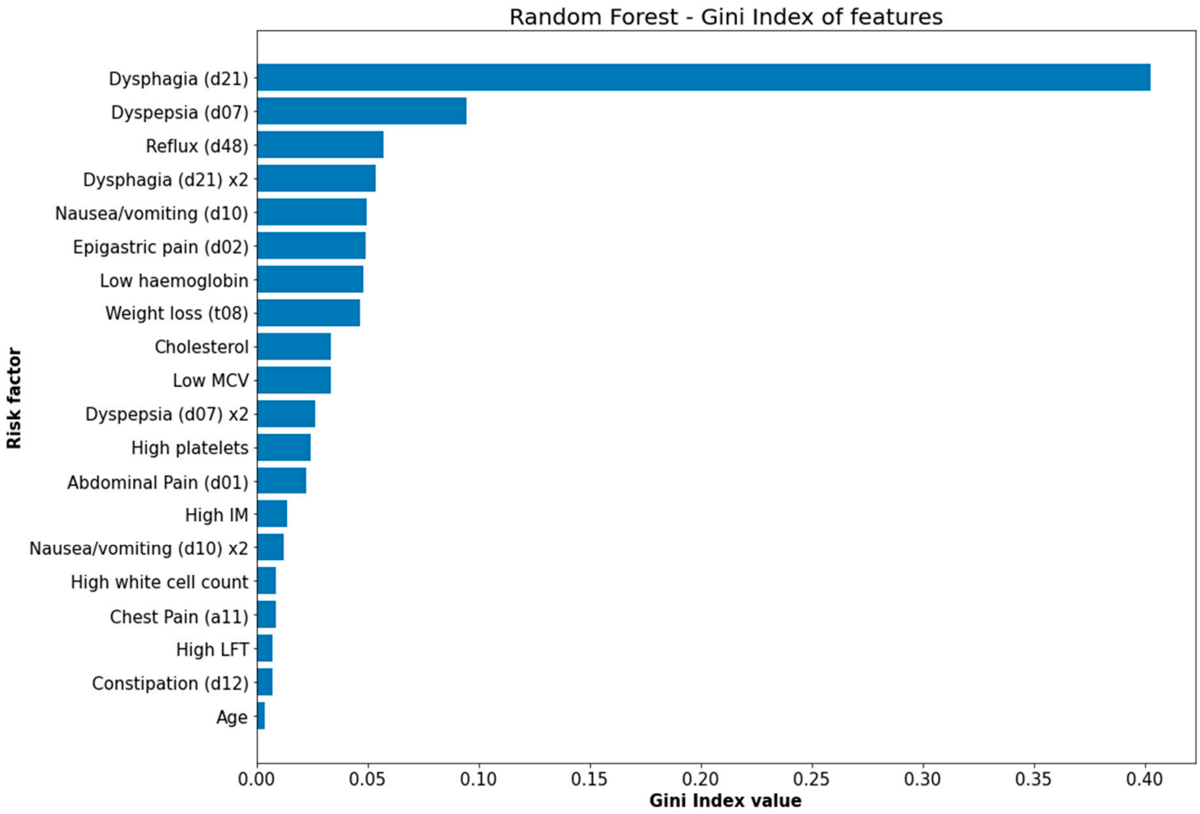

Figure S5. Feature contribution estimation for Random Forest. Feature contribution values correspond to mean decrease in impurity (Gini).

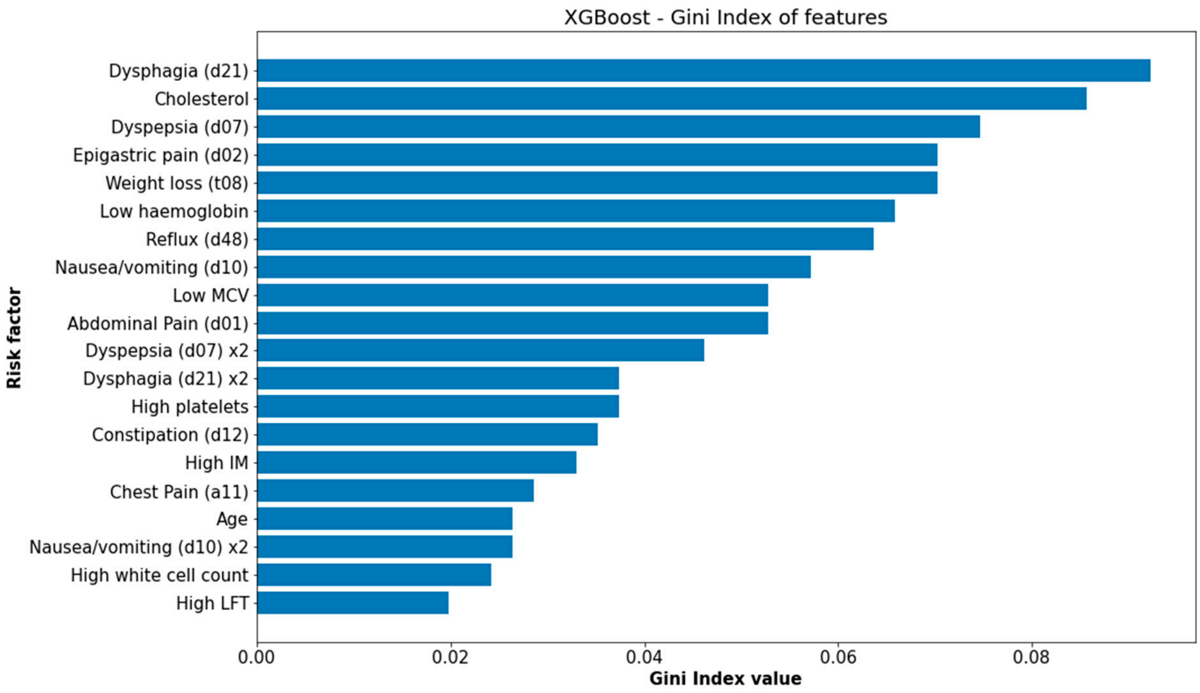

Figure S6. Feature contribution estimation for Extreme Gradient Boosted Decision Trees. Feature contribution values correspond to mean decrease in impurity (Gini).

Table S4. Model performances stratified across different patient groups according to demographics (sex, age group) and cancer site, demonstrated for a selection of some of the best-performing models (linear support vector machine and logistic regression) in comparison to the oesophago-gastric cancer Risk-Assessment Tool (ogRAT). N.B: Since the ogRAT only gives risk scores for over 55s, recall is 0 for the under 55 age group, and precision cannot be calculated.

Model	Variable	Patient subgroup	Accuracy	Precision	Recall
**Linear Support Vector Machine (Classification threshold 0.55)**	**Sex**	Male	0.890	0.832	0.500
		Female	0.900	0.813	0.549
	**Age Group**	Under 55	0.882	0.889	0.559
		55 and over	0.894	0.819	0.514
	**Cancer Site**	Oesophageal	0.909	0.846	0.593
		Gastric	0.864	0.771	0.384
**Logistic Regression (Classification threshold 0.425)**	**Sex**	Male	0.893	0.786	0.568
		Female	0.897	0.768	0.595
	**Age Group**	Under 55	0.890	0.871	0.615
		55 and over	0.894	0.772	0.574
	**Cancer Site**	Oesophageal	0.908	0.796	0.646
		Gastric	0.869	0.740	0.457
**ogRAT (Classification threshold 0.02)**	**Sex**	Male	0.868	0.895	0.312
		Female	0.882	0.900	0.374
	**Age Group**	Under 55	0.769	N/A	0.0
		55 and over	0.879	0.897	0.362
	**Cancer Site**	Oesophageal	0.893	0.920	0.435
		Gastric	0.835	0.797	0.155
**ogRAT (Classification threshold 0.01)**	**Sex**	Male	0.879	0.828	0.424
		Female	0.892	0.834	0.488
	**Age Group**	Under 55	0.769	N/A	0.0
		55 and over	0.891	0.834	0.485
	**Cancer Site**	Oesophageal	0.900	0.850	0.526
		Gastric	0.854	0.777	0.307

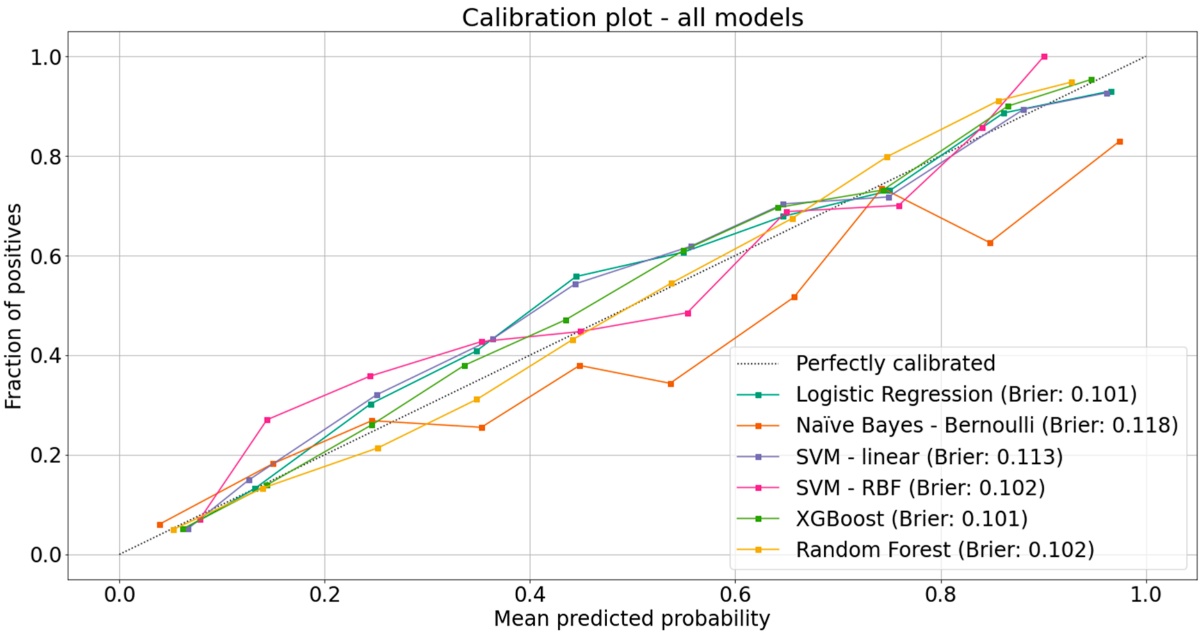

Figure S7. Calibration plot demonstrating the fraction of observed positives in the test dataset across the range of predicted probabilities, and the Brier score, for all models.
SVM – Support Vector Machine. RBF – Radial Basis Function. XGBoost – eXtreme Gradient Boosted decision trees.

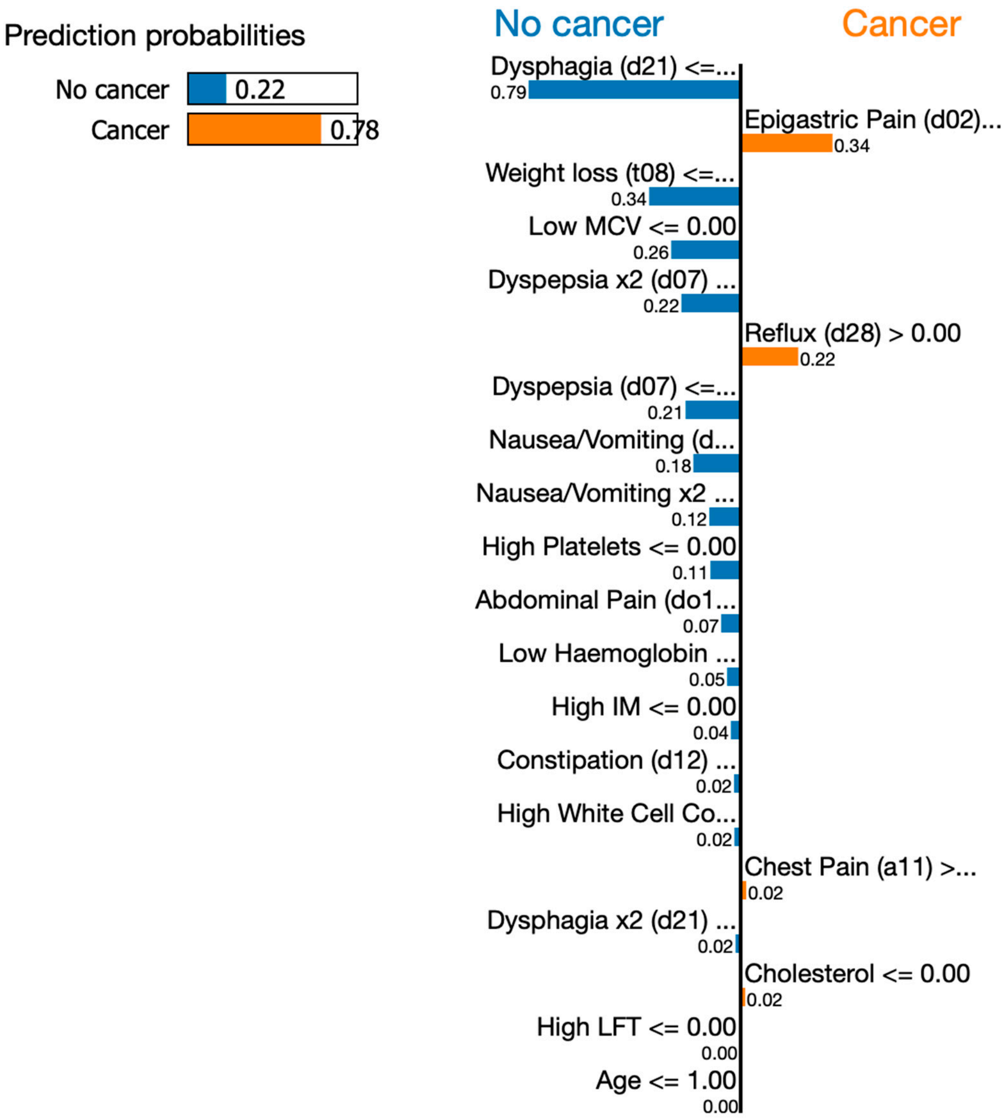

Figure S8. Example of an explanation for an individual prediction, demonstrating a cancer case with vaguer symptoms to which ML-based tools would assign a high risk score, whereas the current oesophago-gastric cancer Risk Assessment Tool (ogRAT) would not. Explanation generated using the Local Interpretable Model-Agnostic Explanations package [38]. Model: Linear Support Vector Machine.

References

Ribeiro, M.T.; Singh, S.; Guestrin, C. 'Why Should I Trust You?': Explaining the Predictions of Any Classifier. In Proceedings of the 22nd ACM SIGKDD International Conference on Knowledge Discovery and Data Mining, San Francisco, CA, USA; ACM: 2016; pp. 1135–1144. Available online: https://dl.acm.org/doi/10.1145/2939672.2939778 (accessed on 12 August 2021).
